# Age- and diet-driven assembly of the gut antibiotic resistome in humans and food-producing animals

**DOI:** 10.1080/19490976.2025.2610052

**Published:** 2026-01-04

**Authors:** Tao Zhang, Jing Wang, Qingying Feng, Xinming Xu, Weiyun Zhu, Shengyong Mao, Jinxin Liu

**Affiliations:** a Laboratory of Gastrointestinal Microbiology, College of Animal Science and Technology, Nanjing Agricultural University, Nanjing, People's Republic of China; b Jiangsu Key Laboratory of Gastrointestinal Nutrition and Animal Health, National Center for International Research on Animal Gut Nutrition, College of Animal Science and Technology, Nanjing Agricultural University, Nanjing, People's Republic of China; c Division of Infectious Diseases, Department of Medicine, Icahn School of Medicine at Mount Sinai, New York, NY, USA; d Institutes of Biomedical Sciences, Fudan University, Shanghai, People's Republic of China; e Department of Nutrition and Food Hygiene, School of Public Health, Institute of Nutrition, Fudan University, Shanghai, People's Republic of China; f Sanya Institute of Nanjing Agricultural University, Nanjing Agricultural University, Sanya, China

**Keywords:** Humans, food-producing animals, antimicrobial resistance, age-dependence, dietary intervention, gut resistome

## Abstract

Antimicrobial resistance (AMR) poses a growing threat to global health, and increasing evidence reveals a substantial overlap in resistance genes between the gut microbiota of humans and food-producing animals, suggesting potential for cross-species transmission. Understanding the early-life development of the gut resistome is essential for designing effective AMR prevention strategies. This review synthesizes current knowledge on the age-dependent assembly of the gut resistome in both humans and food-producing animals, highlighting a consistent pattern of high antimicrobial resistance genes (ARGs) loads at birth followed by a gradual decline with age. We emphasize the critical role of diet in shaping resistome dynamics, formula feeding and high-fat, high-protein diets are associated with increased ARGs burden, whereas breastfeeding and diverse, fiber-rich diets are linked to reduced ARG prevalence. Furthermore, we discuss the potential of probiotics and prebiotics to mitigate gut AMR, while underscoring the importance of assessing resistance gene transfer risk in functional food development. Finally, we outline key knowledge gaps and propose future research directions within the framework of “One Health”. This review provides a comprehensive foundation for policy and intervention strategies to control gut-derived AMR and protect public health.

## 1. Introduction

The widespread use of antibiotics since the mid-20th century has transformed human and veterinary medicine, agriculture, and food production. However, excessive and often indiscriminate use of these agents has accelerated the emergence and spread of antimicrobial resistance (AMR), threatening the effectiveness of standard therapies and contributing to the rise of “multi-drug resistant organisms”.^1-3^ In 2019, AMR was associated with over 1.2 million deaths globally, surpassing the mortality burden of AIDS and malaria.^4^ Without effective intervention, the World Health Organization (WHO) warns that AMR could cause up to 10 million deaths annually by 2050.^5^

Originally proposed by Wright and colleagues in 2007,^6^ the term “resistome” refers to the full repertoire antimicrobial resistance genes (ARGs) within environmental microbial communities and has since been widely adopted in resistance gene research in soil, gut, and diverse ecological niches.^7^ The gastrointestinal tract (GIT) is now recognized as a major reservoir of ARGs in both humans and food-producing animals.^8-10^ Increasing evidence demonstrates substantial overlap in ARG profiles across these populations, facilitating cross-species transfer of ARGs and increasing the risk of zoonotic or environmental dissemination.^11-13^ While antibiotic exposure in clinical or agricultural settings has long been considered the dominant driver of resistome development, emerging research reveals that individuals with no recent antibiotic use may also harbor high levels of ARGs.^14-16^ This observation has prompted growing attention to non-antibiotic factors, including host genetics,^17^ physiological age,^18-20^ diet,^21-23^ and environmental exposures,^24-26^ that shape the resistome.

Among these factors, early-life developmental windows characterized by rapid microbial succession and dietary transitions appear to play a pivotal role in resistome assembly. In this review, we synthesize current knowledge on AMR transmission across humans, animals, and the environment, with a particular focus on age and diet-associated dynamics of the gut resistome. Framed within a “One Health” perspective, our aim is to elucidate the biological and ecological underpinnings of ARG acquisition and dissemination, and to inform targeted strategies for mitigating AMR at the human–animal–environment interface.

## 2. Global dynamics of antimicrobial resistance: transmission across humans, food-producing animals, and the environment

Humans and food-producing animals are central to the global ecology of AMR. As both prescribers and consumers of antibiotics, humans directly influence AMR development and dissemination patterns.^27-29^ Simultaneously, food-producing animals serve as important reservoirs of resistant bacteria and play a significant role in environmental AMR propagation.^30-33^ In particular, the widespread use and often misuse of antibiotics in intensive livestock systems has imposed strong selection pressures on gut microbiota.^30^^,^^34^^,^^35^ Resistant bacteria originating from the GIT of animals are frequently excreted through feces, urine, and other waste, leading to contamination of water, soil, and other environmental reservoirs.^36-41^ These resistant microbes can re-enter the human food chain, thereby promoting cross-species transmission of ARGs.^42^^,^^43^ Recent investigations indicate that most known ARGs are widely disseminated throughout the food production chain. Specifically, these resistance genes are often enriched in food ingredients after processing, particularly in bacterial genera such as *Staphylococcus*, which harbor resistance genes like *tetK* and *fosD*.^44^

Recent metagenomic analyses reveal substantial overlap in ARG profiles between the gut microbiota of humans and food-producing animals, particularly for genes conferring resistance to β-lactams, quinolones, and tetracyclines.^11^^,^^45-49^ Among these, *Escherichia coli* (*E. coli*) is a common and highly relevant host of ARGs in both groups. Genetically similar *E. coli* strains isolated from humans and broiler chickens often harbor identical ARGs-including *CTX-M-55*, *tetM*, and *QnrS1*-transmitted via shared mobile genetic elements (MGEs).^37^^,^^42^^,^^50^ These findings underscore the widespread occurrence of shared ARGs in the gut microbiota of humans and animals and suggest a high potential for interspecies gene flow.

Notably, such ARG transfer events can occur with remarkable speed. A recent longitudinal study showed that even brief exposure to a swine farming environment resulted in rapid remodeling of the human gut resistome. After just three weeks of exposure, the abundance of ARGs associated with β-lactams, quinolones, and tigecycline in the students' gut significantly increased, with levels remaining comparable to those observed in farm workers.^46^ This highlights the ease and speed with which ARGs can disseminate between animal and human populations, especially in the absence of strict biosecurity and antibiotic stewardship measures.

**Figure 1.**
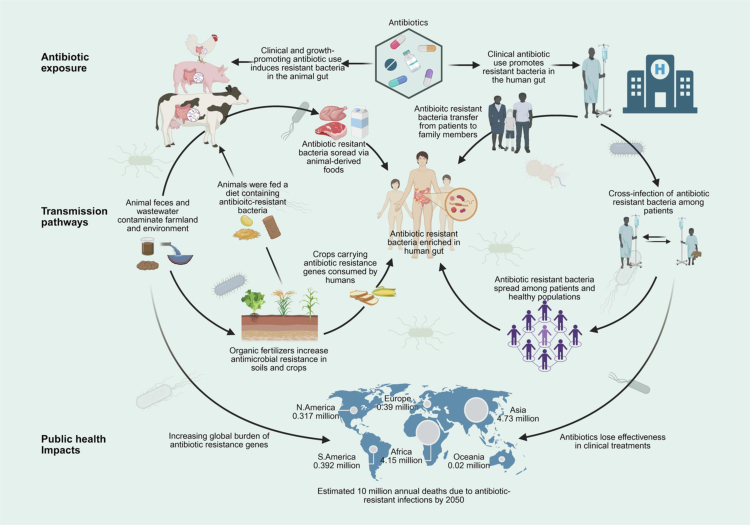
The spread of antibiotic resistance genes endangers human public health security. Antibiotic use in clinical settings and animal husbandry drives the emergence and spread of resistant bacteria across humans, animals, and the environment. Clinical applications facilitate patient-to-patient transmission and community dissemination, while agricultural use introduces resistant strains into the food chain and contaminates crops via manure. Together, these interconnected pathways amplify the global burden of antibiotic resistance, undermining treatment efficacy and threatening public health worldwide.

As our understanding of global AMR deepens, the ecological interdependence of humans, animals, and the environment has become increasingly clear. ARGs frequently move across these domains, forming a complex transmission network (Figure 1).^13^^,^^51^ Mismanagement in any sector can trigger broad and cascading resistance threats. The “One Health” approach has thus gained attention as an integrative framework to track, manage, and reduce ARG dissemination. It emphasizes coordinated surveillance across human, animal, and environmental microbiomes.^52-54^

## 3. Direct and indirect selective pressures drive the amplification of antimicrobial resistance

Antibiotics remain the primary selective force driving the accumulation of AMR in the gut microbiota of both humans and food-producing animals.^55-58^ By targeting bacterial processes-including cell wall synthesis, nucleic acid replication, and protein biosynthesis, antibiotics impose strong ecological pressure that favors the survival and proliferation of resistant strains.^59-62^ In response to the global AMR crisis, many high- and middle-income countries have implemented stricter regulations to reduce clinical and agricultural antibiotic use.^63^^,^^64^ Nevertheless, despite some success in curbing antibiotic consumption,^65^ the prevalence of antimicrobial resistance continues to rise, particularly in gut microbial communities.^66^ This discrepancy highlights that factors beyond direct antibiotic exposure-namely, indirect environmental pressures-also play a crucial role in shaping the resistome.

Increasing evidence points to heavy metals and biocides as important indirect drivers of gut AMR. These agents, commonly used in agricultural, industrial, and healthcare settings, exert selective pressure that can enrich for resistant microbes through mechanisms of co-resistance and cross-resistance.^67-69^ Heavy metals such as copper, zinc, and mercury are widely incorporated into animal feeds and disinfectants. These compounds can facilitate horizontal gene transfer (HGT) by co-selecting for MGEs carrying both metal resistance genes and ARGs.^70-72^ For example, dietary zinc supplementation at high concentrations has been shown to significantly restructure the gut resistome in animals, increasing the abundance of ARGs such as *aph(3”)-Ib*, *pat(A)*, and *lnu(C)*.^73^

Biocides, including quaternary ammonium compounds (QACs) and agricultural pesticides, can similarly promote AMR by selecting for general stress-response mechanisms, such as multi-drug efflux pumps, which confer simultaneous resistance to antibiotics and biocidal agents.^74^ QACs are among the most commonly used disinfectants in clinical settings, particularly with the increased use of QACs, such as benzalkonium chloride (BAC), during the COVID-19 pandemic.^75^ Numerous studies have shown that prolonged exposure to sub-lethal concentrations of benzalkonium chloride not only reduces the susceptibility and enhances the resistance of opportunistic pathogens, such as *Staphylococcus epidermidis* (*S. epidermidis*), *Klebsiella pneumoniae* (*K*. *pneumoniae*), *E. coli*, and *Pseudomonas aeruginosa*, but also promotes the horizontal transfer of extracellular ARGs through natural transformation.^76^ This increases the risk of co-colonization of microbiota with antibiotic resistance genes. Recent evidence from hospital environments using QACs indicates that *Staphylococcus aureus* has developed increased resistance to β-lactam antibiotics.^77^ Furthermore, in an intervention trial targeting *Acinetobacter baumannii* (*A. baumannii*), exposure to BAC prevented the bacterium's susceptibility to gentamicin and significantly increased the frequency of resistance mutations by reducing intracellular antibiotic accumulation.^78^ During this process, BAC also elevated the minimum inhibitory concentration (MIC) of *A. baumannii* to various aminoglycosides, including kanamycin, tobramycin, streptomycin, gentamicin, and amikacin. These findings underscore the unintended consequences of non-antibiotic antimicrobial agents in shaping the gut resistome.

The continued emergence and persistence of antibiotic resistance, even in the absence of high clinical antibiotic exposure, underscores the need for a more holistic approach to AMR mitigation. While prudent antibiotic stewardship remains essential, parallel efforts are needed to limit environmental co-selective pressures. Strategies such as minimizing heavy metal pollution, adopting biocide alternatives with reduced resistance potential, and enforcing stricter regulation of antimicrobial agents in agriculture and industry will be critical to curbing the spread of resistance. Ultimately, controlling both direct and indirect selective pressures is imperative for halting the global rise of antibiotic-resistant bacteria.

## 4. Age-dependent assembly of the gut resistome in early life

While antibiotic and heavy metal exposure significantly contribute to the development of the gut resistome, they are not strictly necessary for its establishment. Emerging evidence indicates that the gut resistome follows a natural, age-dependent assembly trajectory, largely shaped by host developmental and physiological processes, even in the absence of external selective pressures.^14^^,^^16^^,^^79^ This process is particularly pronounced in newborns and young food-producing animals, where early-life colonization events have a profound impact on long-term resistome composition.

The gut resistome begins to assemble immediately after birth.^15^^,^^16^^,^^80^ Numerous recent studies have shown that newborns harbor a diverse and abundant repertoire of ARGs, even in the absence of prior exposure to antibiotics or other stressors.^81-85^ For example, healthy human infants have been found to carry ARGs conferring resistance to β-lactams (e.g., *mecA*) and tetracyclines (e.g., *tetW*) in their fecal microbiota within the first days of life.^86-88^ Similar findings have been reported in food-producing animals.^89^ Gaire et al.^90^ detected ARGs, including those encoding resistance to tetracyclines, aminoglycosides, macrolide-lincosamide-streptogramin (MLS), and β-lactams, in piglets prior to any antibiotic exposure, highlighting an intrinsic potential for early ARG acquisition.

This early ARG burden is largely attributed to colonization by maternal and environmental microbes, especially *E. coli*, a dominant early-life colonizer that frequently carries ARGs. One study found that *E. coli* was the likely host for 36 of the 50 most abundant ARGs detected in the infant gut microbiome.^91^ Notably, when infants were stratified by ARG richness and diversity, *E. coli* abundance was the most discriminating factor. In fact, 94% of the 58 ARGs showing the greatest variability between high- and low-resistance groups were associated with the *E. coli* genome.^86^ These findings suggest that early colonization by *E. coli* plays a pivotal role in shaping the neonatal gut resistome and may offer a valuable target for intervention strategies aimed at mitigating early-life ARG accumulation.

As infants age, the total ARG burden typically declines, coinciding with the maturation of the gut microbiota. ARG abundance in human infants generally peaks at birth and declines throughout the first year of life.^85^^,^^92^^,^^93^ Pärnänen et al.^88^ observed that this decline in ARG abundance was associated with the increasing dominance of low-ARG taxa, such as *Bifidobacterium*, which help stabilize the microbial community and inhibit the persistent invasion of opportunistic pathogens like *Enterococcus faecalis*.^94^^,^^95^ Recent studies have further emphasized the crucial role of *Bifidobacterium* in shaping the infant gut resistome, with its high abundance correlating with a lower ARG burden.^84^^,^^95^^,^^96^ Additionally, in populations with higher *Bifidobacterium* abundance, ARGs exhibit lower transferability, with fewer resistance genes for tetracyclines and β-lactams carried on plasmids.^81^ This pattern of early resistome development has also been observed in food-producing animals. In piglets, calves, and other food-producing species during the suckling period, the gut ARG burden tends to decline gradually with age.^21^^,^^90^^,^^97^ However, the resistome in animals is often more complex and diverse than in humans, likely reflecting more frequent antimicrobial exposure and the broader range of selective pressures encountered under intensive production systems.

Interestingly, the composition of the gut resistome changes not only in magnitude but also in ARG type. In humans, the transition from the neonatal period to early childhood (1–3 years) involves a distinct shift in ARG profiles. Xu et al.^85^ identified two major resistome phases: an early multi-drug resistance phase (0–7 months), followed by a dominance of tetracycline-, mupirocin-, and β-lactam-resistance genes (8–14 months). A similar pattern occurs in food-producing animals. As their gut microbiota matures, the abundance of opportunistic pathogens such as *Enterobacteriaceae,* which are often associated with multi-drug resistance, declines, while more specific ARGs (e.g., those against tetracyclines and MLS) increase in prevalence.^21^^,^^89^

Despite species-specific differences, a conserved pattern emerges: with increasing age, multi-drug resistance tends to decline, while more narrowly targeted ARGs become prevalent. This progression likely reflects both the ecological maturation of the gut microbiome and the host’s cumulative exposure to dietary and pharmacological factors. Table 1 summarizes key age-related resistance genes trends across humans and major food-producing animals.^21^^,^^85^^,^^90^^,^^92^^,^^97-100^

**Table 1.** The dynamic of gut resistome during early life in humans and food-producing animals.

Type	Day of life	Antimicrobial resistance genes changes	Reference
Infants	0−14 month	Increase: Tetracyclines, β-lactams, Aminoglycosides, Mupirocin Decrease: Multi-resistance, Acid_resistance, Arsenic_resistance, Copper_resistance, Nickel_resistance, Peroxide_resistance, Zinc_resistance	[85]
Infants	7−365 d	Increase: β-lactams, Tetracyclines, Macrolide Decrease: Multi-drug resistance, Fluoroquinolone, Rifamycin, Peptide, Diaminopyrimidine	[92]
Pigs	0−179 d	Increase: Tetracyclines, Aminoglycosides, MLS Decrease: Multi-resistance, Copper resistance	[90]
Pigs	0−180 d	Increase: Aminoglycosides, Tetracyclines, Chloramphenicol, MLS Decrease: Multi-drug resistance	[98]
Pigs	0−21 d	Increase: β-lactams, Tetracyclines, MLS Decrease: Multi-drug resistance, Aminoglycosides, Fluoroquinolone, Elfamycins, Bacitracin	[99]
Calf	0−7 weeks	Increase: Tetracycline, MLS Decrease: Multi-drug resistance, β-lactams, Elfamycins, Fluoroquinolones, Cationic antimicrobial peptides, Aminocoumarins	[21]
Goats	1−84 d	Increase: MLS, Aminoglycosides, Tetracyclines Decrease: Multi-resistance, Elfamycins, Copper resistance, Acid resistance, Fluoroquinolones	[97]
Broiler	7−42 d	Increase: Tetracyclines, Aminoglycosides, Carbapenem, MLS, Cephalosporin, Phenicol Decrease: Elfamycin, Glycopeptide, Macrolide, Multi-drug resistance	[100]

## 5. The potential of diet to regulate the gut resistome

Dietary modulation of the gut resistome has recently gained attention as a promising strategy to mitigate AMR, particularly during the critical window of early life. Diet plays a pivotal role in shaping the gut microbiota, which in turn influences the abundance, diversity, and dissemination potential of ARGs. Although research in this area is still limited, emerging evidence suggests that nutritional strategies may offer effective avenues to reduce the burden of ARGs in both humans and food-producing animals. This section synthesizes current insights into how diet influences the gut resistome, with a focus on early-life nutrition, dietary composition, and microbiota-targeted interventions.

### 5.1. Formula feeding is associated with increased ARG burden

During the neonatal period, infants may be breast-fed, formula-fed, or receive a combination of both. Formula feeding is often employed in human infants due to limited human milk availability, while in food-producing animals it is widely adopted for cost efficiency. Multiple studies have demonstrated that formula feeding is associated with a significantly higher gut ARG load compared to breastfeeding.^101-103^

Human milk delivers commensal microbes, such as *Bifidobacterium* and *Lactobacillus*,^104-106^ which are generally low in ARG content and help stabilize the gut ecosystem through competitive colonization.^102^^,^^107^ Although human milk can transmit some resistant bacteria, these are usually associated with low ARG load and are less likely to contribute to resistome amplification. In a mother-infant cohort study, 40% of ARGs and 37% MGEs were shared between infants and their mothers’ gut microbiota, whereas the overlap with human milk was only 20% and 12%, respectively. These results highlight that the role of human milk transmission in shaping the infant gut resistome is minimal.^88^ In contrast, formula feeding has been shown to increase neonatal gut ARG burden by approximately 69%, particularly ARGs conferring resistance to aminoglycosides, β-lactams, and macrolide-lincosamide-streptogramin B (MLSB) antibiotics.^101^ Additionally, all classes of MGEs were found to be more abundant in formula-fed than in breast-fed infants.^102^

**Figure 2.**
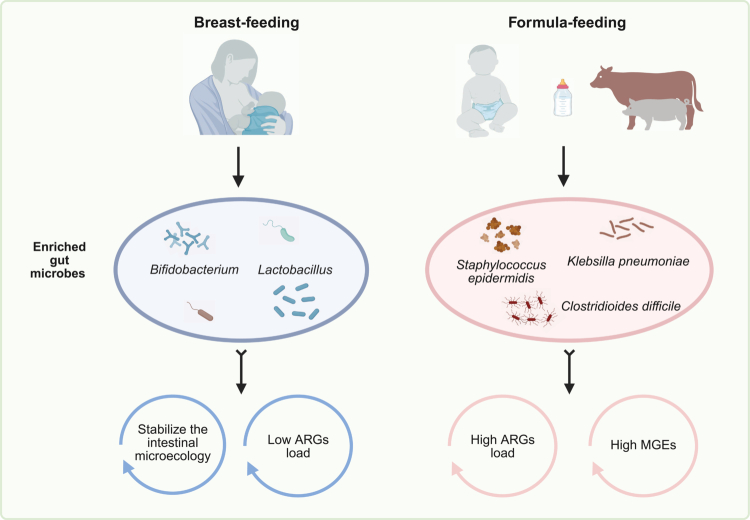
Formula-feeding enhances the accumulation and dissemination of gut antimicrobial resistance genes. Breast-feeding enriches beneficial genera like *Bifidobacterium* and *Lactobacillus*, stabilizing gut ecology and reducing ARG load. In contrast, formula-feeding promotes opportunistic pathogens such as *S. epidermidis*, *K. pneumoniae*, and *C. difficile*, resulting in higher ARG and MGE abundance that facilitates ARG dissemination. ARGs: Antimicrobial resistance genes. MGEs: Mobile gemetic elements.

Formula feeding also promotes the expansion of opportunistic pathogens, such as *S. epidermidis*, *K. pneumoniae*, and *Clostridioides difficile* (*C. difficile*), which act as ARG reservoirs.^101^^,^^108^ Notably, a consistent post-formula increase in *C. difficile* has been observed in both human infants and piglets,^101^^,^^103^ further linking formula feeding to elevated resistome burdens. These findings highlight the protective role of breast-feeding against early-life resistome amplification (Figure 2).

### 5.2. Monotonous diets amplify the gut resistome

The diversity of the diet plays a fundamental role in shaping both microbial diversity and the ecological balance within the gut. Diets lacking variety-particularly those rich in fat, protein, or easily digestible carbohydrates, can disrupt this balance, fostering the accumulation of ARGs.^23^^,^^109-112^ A monotonous diet, defined by a limited range of foods or food groups, can result in nutrient imbalance and negatively impact the gut microbiota. In contrast, diets that are rich in fiber and diverse in composition are associated with lower ARG levels.

**Figure 3.**
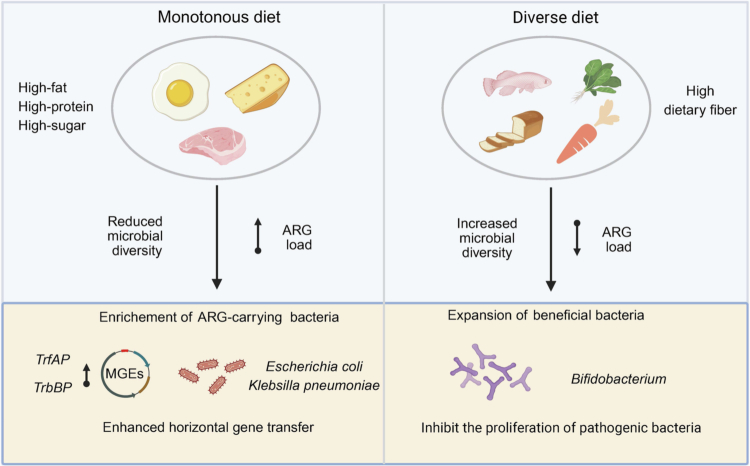
Monotonous high-concentrate diet intensifies the accumulation of gut ARGs. A monotonous diet rich in fat, protein, and sugar reduces microbial diversity, enriches ARG-carrying bacteria such as *E. coli* and *K. pneumoniae*, and enhances HGT mediated by mobile genetic elements (MGEs). In contrast, a diverse, fiber-rich diet increases microbial diversity, promotes the growth of beneficial bacteria like *Bifidobacterium*, and suppresses the proliferation of pathogenic bacteria, thereby mitigating ARG accumulation.

From a microbial ecology perspective, dietary imbalances reduce taxonomic diversity and increase the dominance of ARG-carrying bacteria due to diminished competition (Figure 3). In one dietary intervention targeting obesity, increased intake of non-digestible carbohydrates promoted *Bifidobacterium* enrichment and suppressed *E. coli* and *K. pneumoniae*, resulting in overall reduced ARG abundance.^113^^,^^114^ Furthermore, nutrient-rich diets can also stimulate HGT by increasing intestinal permeability, triggering inflammation, and promoting DNA damage and immune activation.^115-117^ For example, Tan et al.^110^ showed that high-fat, high-sugar, and high-protein diets elevated MGE levels, particularly *trfAP* and *trbBP* in mice, facilitating ARG redistribution and persistence. Among these, high-fat diets exerted the strongest impact.

During early development, both humans and food-producing animals typically transition from a simple to a more complex diet. This transition often coincides with a decline in ARG abundance, likely due to enhanced microbial maturation and reduced dominance of ARG-rich taxa such as *E. coli*.^118^ In our previous study of infant gut resistomes, we observed that as infants' diets transitioned to solid foods, including a variety of carbohydrates, both the host and the gut microbiota required more complex metabolic functions. This dietary shift resulted in the enrichment of microbial taxa capable of metabolizing dietary fibers, such as *Bacteroidetes*, which are typically associated with smaller ARG pools. As *Bacteroidetes* became more dominant, taxa such as *Pseudomonadota*, known for their high ARG content, were gradually suppressed, leading to a reduction in the overall ARG burden within the gut.^85^

Similarly, in a study of dairy calves, changes in diet had a profound significant impact on the development of the gut resistome. As calves transitioned from milk to solid feed, the nutritional composition shifted from lactose to starch. This dietary change favored the enrichment of *Bacteroidaceae*, which are rich in amylase and often carry tetracycline-resistant genes like *tetQ*.^21^ This shift accounts for the observed increase in the abundance of certain specific ARG types (e.g., tetracyclines, aminoglycosides, and MLS), despite the general decline in the overall abundance of ARGs during early life. This study also demonstrated that the relative abundance of *Enterobacteriaceae* and other multi-drug resistant taxa, whose decline predominated during this period, was a major factor driving the reduction in total ARG burden.^21^

Accordingly, existing evidence strongly links the transition from a monotonous to a complex diet with the development of ARGs, with diverse diets proving more effective in managing gut resistomes during early life. However, despite promising indications, systematic studies on this relationship remain limited, and the underlying mechanisms are still not well understood.

### 5.3. Probiotics and related interventions to reduce gut ARGs

**Figure 4.**
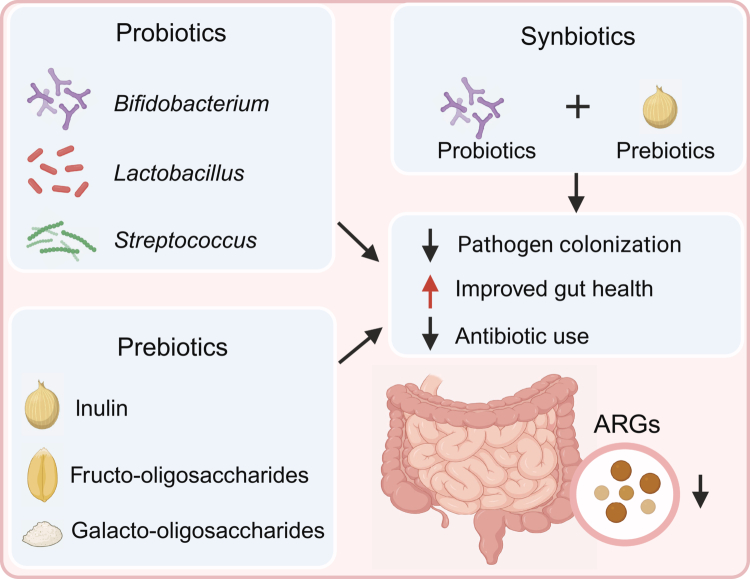
Strategies and mechanisms of probiotics, prebiotics, and synbiotics in limiting the gut resistome. Microbial agents reduce the enrichment of gut ARGs by suppressing pathogen colonization, enhancing gut health, and minimizing antibiotic usage. ARGs: antimicrobial resistance genes.

Microbiota-targeted nutritional interventions, including probiotics, prebiotics, synbiotics, and postbiotics, offer promising avenues for GIT resistome modulation, especially during early life.^119^^,^^120^ These compounds improve gut health by enhancing nutrient utilization, promoting beneficial microbes, and suppressing ARG-carrying pathogens (Figure 4).

Probiotics, defined as live microorganisms that confer health benefits upon ingestion, include strains of *Lactobacillus*, *Bifidobacterium*, *Saccharomyces*, and *Bacillus*.^121-126^ Their benefits include preventing infections, improving gut barrier function, and supporting microbial homeostasis.^127^^,^^128^ Probiotic supplementation during early life is especially effective due to the immature, more receptive gut ecosystem.^129^ Studies show that probiotic use significantly reduces gut ARG diversity in infants, particularly those encoding antibiotic inactivation enzymes (e.g., β-lactamases, aminoglycoside-modifying enzymes).^130^

Prebiotics are non-digestible carbohydrates that selectively stimulate beneficial gut microbes.^131^ Common types include galacto-oligosaccharides, inulin, and fructo-oligosaccharides.^132^ Prebiotics enhance the growth of low-ARG-containing taxa, improve intestinal function, and suppress resistant pathogens like *E. coli* and *K. pneumoniae*.^133-136^ During early life, prebiotic supplementation accelerates gut microbial maturation and promotes a more stable, less resistant community structure.^137^^,^^138^

Synbiotics, combinations of probiotics and prebiotics, offer synergistic benefits by facilitating colonization of beneficial microbes and enhancing substrate utilization.^139^ Previous studies have shown that the effects of probiotics or prebiotics alone are highly individual-specific, potentially limited due to the lack of available substrates or degradable microbial populations.^140-142^ In contrast, synbiotics, by combining probiotics and prebiotics, effectively overcome this limitation. Synbiotics often outperform individual components in promoting gut health and lowering ARG burden.^140^

Despite these benefits, the use of probiotics and related products is not without risks. The effectiveness of probiotics depends on host compatibility and strain colonization potential.^142^ Importantly, some probiotic strains naturally carry ARGs, posing a risk of horizontal transfer.^143-145^ Charteris et al.^146^ found that all 46 tested *Lactobacillus* strains were resistant to at least one antibiotic class, including cefoxitin and gentamicin. Additionally, strains such as *Enterococcus* and *Clostridium perfringens*, isolated after dietary fiber supplementation, showed resistance to penicillin G.^147^ These findings underscore the need for careful screening to exclude strains carrying mobile ARGs during probiotic development.

## 6. Future perspectives

The overuse and misuse of antibiotics have accelerated the establishment and proliferation of the gut resistome. ARGs originating from clinical, agricultural, and environmental sources can accumulate in natural ecosystems and subsequently enter the human and animal GIT through the food chain, water systems, or direct contact. Within the gut microbiota, these ARGs are propagated via both vertical inheritance and HGT, enabling widespread dissemination of resistance traits and complicating the treatment of infectious diseases. Addressing this challenge demands a comprehensive understanding of resistome development from a “One Health” perspective, integrating human, animal, and environmental health to inform more effective surveillance, regulation, and intervention strategies.

Despite advances in high-throughput metagenomic sequencing, our understanding of the ecological and mechanistic underpinnings of resistome assembly and transmission remains incomplete.^148^ In particular, while metagenomic technologies offer broad resistome profiling, they are constrained by database completeness and annotation accuracy, limiting the ability to trace specific gene transfer events.^149^^,^^150^ Emerging long-read sequencing platforms,^151^^,^^152^ such as single-molecule real-time (SMRT) sequencing,^153^ offer improved genome assembly and structural resolution over conventional short-read next-generation sequencing (NGS) methods,^154^ enabling more accurate identification of ARG-host associations.

Complementary approaches such as real-time quantitative PCR and targeted metagenomics provide higher specificity and sensitivity for detecting ARGs and MGEs,^155^ though at the cost of lower genome-wide coverage. Additionally, fluorescent reporter systems have been developed to trace ARG transfer in vitro and in vivo,^156^ but these approaches are contingent on microbial cultivation, which limits their application in uncultured taxa. Therefore, future studies should integrate multi-omics strategies combining metagenomics, metatranscriptomics, single-cell genomics, and metabolomics at the strain level to trace the origins and transfer routes of ARGs, discover novel resistance mechanisms, and characterize their ecological drivers.

Technological innovations in resistome surveillance pave the way for novel control strategies, particularly those targeting the diet–microbiota–resistome axis. Dietary interventions, including functional foods, prebiotics, and synbiotics have shown potential in reducing gut ARG burdens by modulating microbiome composition and functionality. However, the safety of these interventions must be rigorously evaluated to ensure that they do not serve as unintended vectors for ARG dissemination. In particular, microbial products used in functional foods should be screened to exclude strains harboring mobile or transferable ARGs. Furthermore, considering the substantial inter-individual variability in gut microbiota and resistome composition, precision nutrition and personalized dietary interventions should be prioritized in both clinical and agricultural settings. Stratifying individuals or animal populations into responsive cohorts based on microbiome or resistome profiles may enable more effective, targeted modulation of ARG reservoirs.

Beyond dietary strategies, addressing AMR requires systemic, cross-sectoral efforts. Policies must promote the rational use of antibiotics in both human and veterinary medicine, coupled with robust antibiotic stewardship programs and education initiatives to minimize unnecessary administration of broad-spectrum agents. Agricultural practices should transition toward sustainable models that reduce reliance on prophylactic antibiotics, favoring alternatives such as vaccination, microbiome-based approaches, and biosecurity improvements.

In conclusion, resolving the global AMR crisis necessitates an integrative One Health framework that leverages advancements in microbiome science, precision nutrition, and environmental stewardship. Coordinated actions across disciplines will be essential to curb the spread of ARGs and safeguard the efficacy of antibiotics for future generations.

## 7. Conclusion

The emergence and persistence of antibiotic resistance within the gut microbiome pose a critical threat to global health, food security, and medical practice, necessitating urgent and innovative mitigation strategies. Through a “One Health” framework, this review integrates current evidence on the early-life establishment of the gut resistome in both humans and food-producing animals, underscoring diet as a pivotal and modifiable determinant of resistome dynamics. Nutritional strategies such as human milk feeding, dietary diversification, and microbiota-targeted approaches show potential in limiting the enrichment and dissemination of ARGs, particularly during critical developmental periods. Incorporating evidence-based dietary strategies into AMR prevention and control initiatives may enable early mitigation of resistome accumulation at the population level. Nevertheless, findings to date remain heterogeneous, and the persistence and generalizability of beneficial effects require validation through rigorously designed longitudinal and interventional studies supported by advanced multi-omics analyses. Positioning early-life nutritional management as a core component of “One Health” implementation could enhance antibiotic stewardship and contribute meaningfully to reducing AMR risks across human, animal, and environmental domains.
